# Thyroid hormone levels and BMI-SDS changes in adolescents with obesity

**DOI:** 10.3389/fendo.2023.1304970

**Published:** 2023-12-11

**Authors:** Daniela Staníková, Lea Krajčovičová, Denisa Lobotková, Eva Vitariušová, Ľubica Tichá, Zuzana Pribilincová, Barbara Ukropcová, Jozef Ukropec, Juraj Staník

**Affiliations:** ^1^ Department of Pediatrics, Medical Faculty of Comenius University and National Institute for Children´s Diseases, Bratislava, Slovakia; ^2^ Department of Metabolic Research, Institute of Experimental Endocrinology, Biomedical Research Center, Slovak Academy of Sciences, Bratislava, Slovakia; ^3^ Institute of Pathophysiology, Medical Faculty of Comenius University, Bratislava, Slovakia

**Keywords:** adolescents, children, obesity, thyroid hormones, FT4, TSH, BMI-SDS, weight gain

## Abstract

**Background:**

Thyroid hormones play an important role in energy metabolism and weight control, explained mostly by inducing thermogenesis and increasing basal metabolic rate. It has recently been shown that FT4 levels are associated with food preferences, which might also play a role in modulating body weight. The aim of this longitudinal follow-up study was to analyze the relationship of thyroid hormones levels (FT4, TSH) at baseline with weight/BMI-SDS changes in children and adolescents with obesity.

**Methods:**

Three hundred seventy-seven children and adolescents have been enrolled to this study and followed up without a systematic intervention program for 5.59 ± 1.85months. Children and adolescents were divided into three subgroups: 1) 144 adolescents with obesity (15-19 years), 2) 213 children with obesity (10-14.9 years), and 3) 20 lean adolescents (15-19 years). Thyroid hormones were measured at the baseline, and anthropometry was performed at the baseline and during the follow-up. For further analyses, participants were divided according to the BMI-SDS change into two groups: 1. with BMI-SDS decrease, and 2. with BMI-SDS increase.

**Results:**

Adolescents with obesity from the BMI-SDS decrease group had significantly lower baseline serum levels of TSH compared to the BMI-SDS increase group (2.4 ± 1.0 *vs*. 3.2 ± 2.0mIU/l; p=0.005). Similar difference was found for FT4 levels (14.7 ± 2.2 in the BMI-SDS decrease group *vs*. 15.5 ± 2.7pmol/l in the BMI-SDS increase group, p=0.048). Moreover, the BMI-SDS decrease was present in significantly higher percentage of adolescents with obesity with lower than median TSH level compared to those with higher than median TSH level at baseline (61.1% *vs* 38.6%, p=0.011). Likewise, the BMI-SDS decrease was present in significantly higher percentage of adolescent females with obesity and lower than median FT4 compared to those with higher than median FT4 level at baseline (70.6% *vs*. 23.5%, p<0.001). No associations of baseline thyroid hormones with the BMI-SDS change were observed in children with obesity or lean adolescents.

**Conclusion:**

Adolescents with obesity and increased BMI-SDS during the follow-up had significantly higher baseline levels of both TSH and FT4 compared to BMI-SDS decrease group. These results support the previous findings that higher FT4 in individuals with obesity may influence weight gain.

## 1 Introduction

Obesity in children and adolescents is associated with several metabolic and endocrine disorders, including dysregulation of the thyroid axis. However, etiology, pathogenesis and physiological significance of these changes is still not fully understood. It is assumed that altered thyroid function is the consequence rather than the cause of the excess body fat, but the possibility cannot be excluded that it might also contribute to problems with both weight reduction and weight loss maintenance (1).

The most common finding in individuals with obesity and dysregulation of the thyroid axis is increased concentration of thyroid-stimulating hormone (TSH) (2). Reported figures on prevalence of hyperthyrotropinemia (serum TSH level above 4 mIU/L) in children and adolescents with overweight or obesity vary from 2% to 22% (1, 3–5). The majority of studies reported no significant alterations in baseline levels of free thyroxine (FT4) and free triiodothyronine (FT3), however, many of them showed that the levels were higher in children and adolescents with obesity rather than in eutrophic children and adolescents. Some changes have also been observed in the ratio between FT4 and FT3, though within the normal range (2, 6).

Several possible explanations for the elevated TSH levels in people with obesity have been discussed in the literature, with leptin being the most likely link between thyroid function and weight status. Leptin is predominantly released by adipocytes and stimulates thyrotropin releasing hormone (TRH) and TSH secretion by hypothalamic-pituitary axis (7). Simultaneously, leptin modulates the responsiveness of the thyroid gland to TSH and influences the activity of the deiodinases. Inflammatory adipokines produced in the adipocytes in obesity may also alter the activity of deiodinases as well as thyroid function, contributing to morphological and functional changes in the thyroid gland due to chronic low-grade inflammation (2). In addition to thyroid, the presence of TSH receptors was reported in some non-thyroidal tissues including liver and adipocytes, suggesting the potential role of TSH in affecting thermogenesis, adipogenesis and lipolysis/lipogenesis balance (8). Obesity-associated insulin resistance is often linked to the reduced sensitivity to thyroid hormones (9). Finally, thyroid dysfunction may represent simply an adaptation process with the aim to increase energy expenditure (10). Thus, the increase in serum TSH levels observed in individuals with obesity could represent a compensatory mechanism with the aim to minimize weight gain.

This assumption is justified by the role of thyroid hormones in the acceleration of energy metabolism and ATP turnover, especially in the induction of thermogenesis by stimulating the expression and activity of mitochondrial uncoupling protein (UCP). It is worth mentioning that resting energy expenditure indeed rises in obesity, probably due to the concomitant increase in body weight and fat-free mass. However, studies conducted in euthyroid individuals with obesity revealed no association between energy expenditure and concentrations of serum TSH, and FT4 and FT3. One possible explanation is the impairment of thyroid hormones capacity to induce thermogenesis in obesity, due to reduced expression of thyroid hormone receptors, D2 and D3 enzymes, β2 and β3 adrenergic receptors, and UCP-2 in the adipose tissue (especially the visceral) of these individuals (6).

Number of cross-sectional and longitudinal studies have described associations between thyroid hormones and anthropometric parameters in obesity (6). The key role of thyroid hormones in energy metabolism and weight control is generally explained by increasing the basal metabolic rate. thermogenesis, increased lipolysis and catabolism of proteins (11). However, there are few studies indicating that thyroid hormones might modulate the food preferences (12–14), which could also have weight-modulating effect. In our previous study (12), we analyzed interrelations between food preferences and thyroid hormone levels and we have found, that children and adolescents with obesity have different thyroid hormone levels and different food preferences in comparison to those without obesity. Moreover, we found that free thyroxine (TF4) level was positively correlated with preference for a high fat and high protein diet (12). Based on these findings, we hypothesized, that individuals with higher baseline levels of FT4 (indicating a preference for a high fat and high protein diet), would gain more weight in comparison to those who have reached lower levels of FT4 in the 5-month follow up.

Therefore, we aimed to analyze the relationship of baseline thyroid hormone levels with weight changes during the 5-month follow-up in children and adolescents with obesity.

## 2 Materials and methods

### 2.1 Study population

Here we examined interrelations between thyroid hormone levels and BMI-SDS change in a longitudinal 5-month follow-up of 377 children and adolescents from the Endocrinology outpatient clinic of the National Institute for Children’s Diseases in Bratislava. Participants were enrolled during the years 2017-2023, and followed up 5.59 ± 1.85 months. Included were 144 adolescents with obesity aged 15-19 years (study group), 213 children with obesity aged 10-15 years, and 20 adolescents without obesity aged 15-19 years with compensated thyroid disorder or healthy, referred to pediatric endocrinologist with a suspicion of thyroid disease (non-obese control group). Individuals with any other chronic or acute metabolic disorder including diabetes mellitus, and individuals with genetic syndromes were excluded. Participants with incomplete data and participants of the systematic intervention programs have been also excluded. Participants did not have any specific medical-based dietary recommendations. At the time of the baseline examination, anthropometric data were recorded, and blood was sampled for biochemical analysis while a subgroup of children and adolescents with or without the assistance of their parents/guardians filled out the food preference questionnaire. Individuals with obesity received recommendation of rational food and regular exercise, but were not enrolled to the systematic intervention program during the follow-up. At the end of the follow-up anthropometric data were recorded.

### 2.2 Anthropometry

Anthropometric measurements (body height and weight) were taken by trained nurses according to standardized protocols. Body mass index (BMI) was calculated as weight divided by the square of body height. Standard deviation score (SDS) for BMI was calculated using local reference values (15). Categories for the BMI-SDS score were defined as follows: children and adolescents without obesity as the BMI-SDS <1.88, and with obesity as BMI-SDS ≥ 1.88. BMI-SDS was evaluated both, at the baseline and at the end of follow-up, and the BMI-SDS change was calculated. For further analyses, participants were in each of the groups (adolescents with obesity, children with obesity, and adolescents without obesity) divided according to the BMI-SDS change into two subgroups: 1. “weight loss” with a decrease of BMI-SDS, and 2. “weight gain” with the BMI-SDS increase/no change.

### 2.3 Biochemical analyses

Blood for hormonal and biochemical analyses was collected after an overnight fasting in serum tubes between 7.30 and 10.00 a.m. The samples were routinely processed and analyzed by the clinical service laboratory at the National Institute for Children´s Diseases. Thyroid hormone levels and biochemical markers describing the metabolic health in individuals with obesity were selected for the analyses (**Table 1**). Participants in the study group of adolescents with obesity were further stratified to low and high free thyroxine (FT4) and thyroid-stimulating hormone (TSH) subpopulations according to the median level of FT4 and TSH.

**Table 1:** Basic characteristics of the study groups.

Parameter	Obese (15-19 years)	Obese (10-15 years)	p	Lean (15-19 years)	p
*Baseline data*					
**Age (years)**	16.59 ± 0.93 (144)	12.71 ± 1.37 (213)	**<0.001**	16.53 ± 0.98 (20)	0.765
**Sex (% of females)**	47.2 (144)	47.4 (213)	1,000	55.0 (20)	0.634
**Weight (kg)**	102.82 ± 21.63 (144)	83.68 ± 19.64 (213)	**<0.001**	66.3 ± 13.07 (20)	**<0.001**
**Height (cm)**	173.14 ± 9.5 (144)	162.7 ± 10.68 (213)	**<0.001**	171.72 ± 10.49 (20)	0.54
**Height SDS**	0.07 ± 1.08 (144)	0.69 ± 1.19 (213)	**<0.001**	0.01 ± 1.24 (20)	0.816
**BMI (kg/m^2^)**	34.19 ± 6.19 (144)	31.34 ± 5.31 (213)	**<0.001**	22.31 ± 2.69 (20)	**<0.001**
**BMI SDS**	4.94 ± 2.3 (144)	4.21 ± 1.76 (213)	**0.001**	0.54 ± 0.98 (20)	**<0.001**
TSH (mU/l)*	2.46; 1.68-3.42 (142)	2.86; 2.16-3.93 (211)	**0.034**	2.18; 1.63-2.55 (20)	0.117
**FT4 (pmol/l)**	15.07 ± 2.5 (144)	15.46 ± 2.56 (213)	0.157	16.08 ± 2.27 (20)	0.090
**FT3 (pmol/l)**	6.18 ± 1.11 (68)	6.78 ± 0.97 (116)	**<0.001**	6.54 ± 1.13 (9)	0.362
Fasting serum glucose (mmol/l)*	4.80; 4.5-5.1 (128)	5.00; 4.7-5.2 (175)	0.123	5.05; 4.7-5.2 (14)	0.590
**Urea (mmol/l)**	3.85 ± 0.93 (111)	3.92 ± 0.87 (164)	0.511	4.02 ± 1.09 (9)	0.595
**Creatinine (mmol/l)**	63.63 ± 10.8 (137)	52.79 ± 9.8 (193)	**<0.001**	61.24 ± 10.98 (17)	0.391
**Uric acid (μmol/l)**	381.07 ± 89.09 (134)	353.34 ± 80.12 (186)	**0.004**	276.29 ± 79.77 (14)	**<0.001**
**Total serum proteins (g/l)**	72.92 ± 4.93 (43)	73.21 ± 4.14 (76)	0.733	73.4 ± 3.32 (4)	0.851
AST (μkat/l)*	0.4; 0.33-0.49 (132)	0.4; 0.33-0.47 (184)	0.976	0.34; 0.31-0.37 (16)	0.067
ALT (μkat/l)*	0.45; 0.31-0.71 (135)	0.38; 0.28-0.55 (194)	0.144	0.25; 0.2-0.385 (17)	0.073
GGT (μkat/l)*	0.325; 0.26-0.49 (56)	0.25; 0.19-0.31 (101)	**0.006**	0.17; 0.17-0.17 (1)	0.334
ALP (μkat/l)*	1.57; 1.32-1.98 (80)	3.81; 2.59-4.75 (142)	**<0.001**	1.59; 1.13-3.25 (9)	0.477
**Total-cholesterol (mmol/l)**	4.16 ± 0.92 (128)	4.11 ± 0.79 (184)	0.621	3.73 ± 0.57 (13)	0.105
**HDL-cholesterol (mmol/l)**	1.12 ± 0.25 (119)	1.13 ± 0.23 (169)	0.755	1.42 ± 0.25 (6)	**0.006**
**LDL-cholesterol (mmol/l)**	2.68 ± 0.73 (118)	2.62 ± 0.71 (169)	0.496	2.31 ± 0.43 (6)	0.228
**Triglycerides (mmol/l)**	1.31 ± 0.59 (128)	1.38 ± 0.66 (186)	0.307	0.83 ± 0.45 (12)	**0.006**
**Insulin (mU/l)**	30.62 ± 25.41 (81)	31.55 ± 32.66 (126)	0.828	13.85 ± 1.48 (2)	0.356
**High Sugar score**	5.41 ± 1.16 (12)	5.39 ± 1.45 (14)	0.97	6.09 ± 1.01 (12)	0.142
**High Complex Carbohydrate score**	5.68 ± 0.96 (12)	5.67 ± 1.25 (14)	0.985	5.97 ± 1.07 (12)	0.491
**High Protein score**	5.41 ± 1.6 (12)	5.1 ± 1.41 (14)	0.605	5.5 ± 0.97 (12)	0.873
**High Fat score**	5.46 ± 1.01 (12)	5.52 ± 1.35 (14)	0.889	5.7 ± 0.76 (12)	0.511
**Low Fiber score**	5.67 ± 1.43 (9)	5.28 ± 1.61 (9)	0.592	6.05 ± 0.9 (8)	0.535
**Score for foods cotaining ≥1.5g of Saturated FA per 100g**	5.46 ± 1.13 (9)	5.06 ± 1.44 (9)	0.515	6.11 ± 0.67 (8)	0.181
**Score for foods cotaining ≥1.5g of Polysaturated FA per 100g**	5.54 ± 1.07 (9)	5.06 ± 1.42 (9)	0.434	6.2 ± 0.68 (8)	0.16
**Score for foods cotaining ≥1.5g of Monosaturated FA per 100g**	5.54 ± 1.06 (9)	5.13 ± 1.38 (9)	0.493	6.03 ± 0.74 (8)	0.287
*Follow-up data*					
**BMI (kg/m2)**	34.09 ± 6.57 (144)	31.17 ± 5.35 (213)	**<0.001**	22.43 ± 2.69 (20)	**<0.001**
**BMI SDS**	4.89 ± 2.44 (144)	4.08 ± 1.8 (213)	**0.001**	0.58 ± 0.98 (20)	**<0.001**
**Duration of the follow-up (months)**	5.40 ± 1.92 (144)	5.76 ± 1.80 (213)	0.096	6.60 ± 1.92 (20)	**0.007**
**BMI change**	-0.1 ± 2.23 (144)	-0.17 ± 2.24 (213)	0.773	0.12 ± 0.84 (20)	0.407
**BMI-SDS change**	-0.05 ± 0.83 (144)	-0.13 ± 0.78 (213)	0.334	0.04 ± 0.31 (20)	0.362
**BMI-SDS change per year**	-0.18 ± 2.02 (144)	-0.29 ± 1.79 (213)	0.578	0.06 ± 0.49 (20)	0.237

*Non-normally distributed data (TSH, fasting serum glucose, AST, ALT, GGT and ALP) are presented as the median and interquartile range, and were log transformed prior t-test. TSH, Thyroid-stimulating hormone; FT4, free thyroxine; FT3, free triiodothyronine; BMI, body mass index; SDS, standard deviation score; AST, aspartate aminotransferase; ALT, alanine aminotransferase; GGT, gamma-glutamyl transferase; ALP, alkaline phosphatase; FA, fatty acids. Value in parentheses (number of participants). P value was calculated in t-test for the difference between populations of adolescents with obesity and 1. children with obesity and 2. non-obese adolescents. All significant values (p<0.05) are in bold.

### 2.4 Assessing food preferences

Food preferences for a subgroup of participants were assessed using the validated Food preference questionnaire (16). The food preference questionnaire requires patients to rate 72 food items on a 9-point scale ranging from “dislike a lot” (1-point), neutral feeling about the food (5-points) to “like a lot”(9-points). If patients did not have a memory of trying the particular food item or if they have never tested it, “I don´t know” was selected. Food items were classified into 12 groups according to nutrient composition; 8 of them were used in this study (high sugar score, high complex carbohydrate score, high protein score, high fat score, high saturated fatty acids score, high monounsaturated fatty acids score, high polyunsaturated fatty acids score, and low dietary fibers score).

### 2.5 Statistics

Variables were checked for normality using the Shapiro–Wilk test. Normally distributed data are expressed as the mean ± SD. Non-normally distributed data (TSH, glucose, aspartate aminotransferase - AST, alanine aminotransferase - ALT, gamma-glutamyl transferase - GGT, and alkaline phosphatase - ALP) are presented as the median and interquartile range, and were logarithmically transformed prior further analyses. Confidence intervals for percentages in binary data were calculated using the Wilson/Brown method. Differences between the two groups were tested using the two-sided Student’s t-test, and by Fisher’s test for binary data. Multivariate associations between selected variables were determined in forward logistic multiple regression analyses. Change of the BMI-SDS coded as a binary variable (i.e. 0 for BMI-SDS increase/no change, and 1 for BMI-SDS decrease) was used as dependent variable, and age, sex, FT4 and TSH as co-variates. P<0.05 was considered statistically significant. Statistical analyses were performed using the SPSSv27 (IBM, USA), JMP (USA) and GraphPad Prism 7 (GraphPad, USA) software.

### 2.6 Ethics committee

The study was approved by the Ethics Committee of the National Institute for Children´s Diseases in Bratislava, Slovakia and adhered to the tenets, outlined in the declaration of Helsinki, modified in 2013. A written informed consent was obtained from the parents; juveniles gave verbal assent.

## 3 Results

### 3.1 Study population

This longitudinal study included 377 children and adolescents; 144 adolescents with obesity (76 boys/68 girls, mean age16.59 ± 0.93 years, mean BMI-SDS 4.94 ± 2.3), group of 213 children with obesity (112 boys/111 girls, mean age 12.71 ± 1.37 years and mean BMI-SDS 4.21 ± 1.76) and 20 adolescents without obesity (9 boys/11 girls, mean age 16.53 ± 0.98 years and mean BMI-SDS 0.54 ± 0.98). Compared to children with obesity, adolescents with obesity had significantly lower levels of TSH (median 2.46 and interquartile range of 1.68-3.42 *vs* 2.86; 2.16-3.93 mU/l; p=0.034) and FT3 (mean 6.18 ± 1.11 *vs*. 6.78 ± 0.97 pmol/l, p<0.001). Differences between the study groups were also seen in the serum levels of uric acid, gamma-glutamyl transferase, and alkaline phosphatase (**Table 1**). The duration of the follow up did not differ significantly between adolescents and children with obesity (0.45 ± 0.16 *vs*. 0.48 ± 0.15 years, p=0.096); follow-up of non-obese adolescents was significantly longer (0.45 ± 0.16 *vs*. 0.55 ± 0.16 years, p=0.007). There were no significant differences between the groups in the BMI or BMI-SDS changes during the follow-up (**Table 1**).

### 3.2 BMI, weight changes and thyroid hormones

For further analyses, we divided participants in each group into two subgroups based on the BMI-SDS change: 1. a decrease of BMI-SDS, and 2. an increase/no change of BMI-SDS.

**Figure 1:**
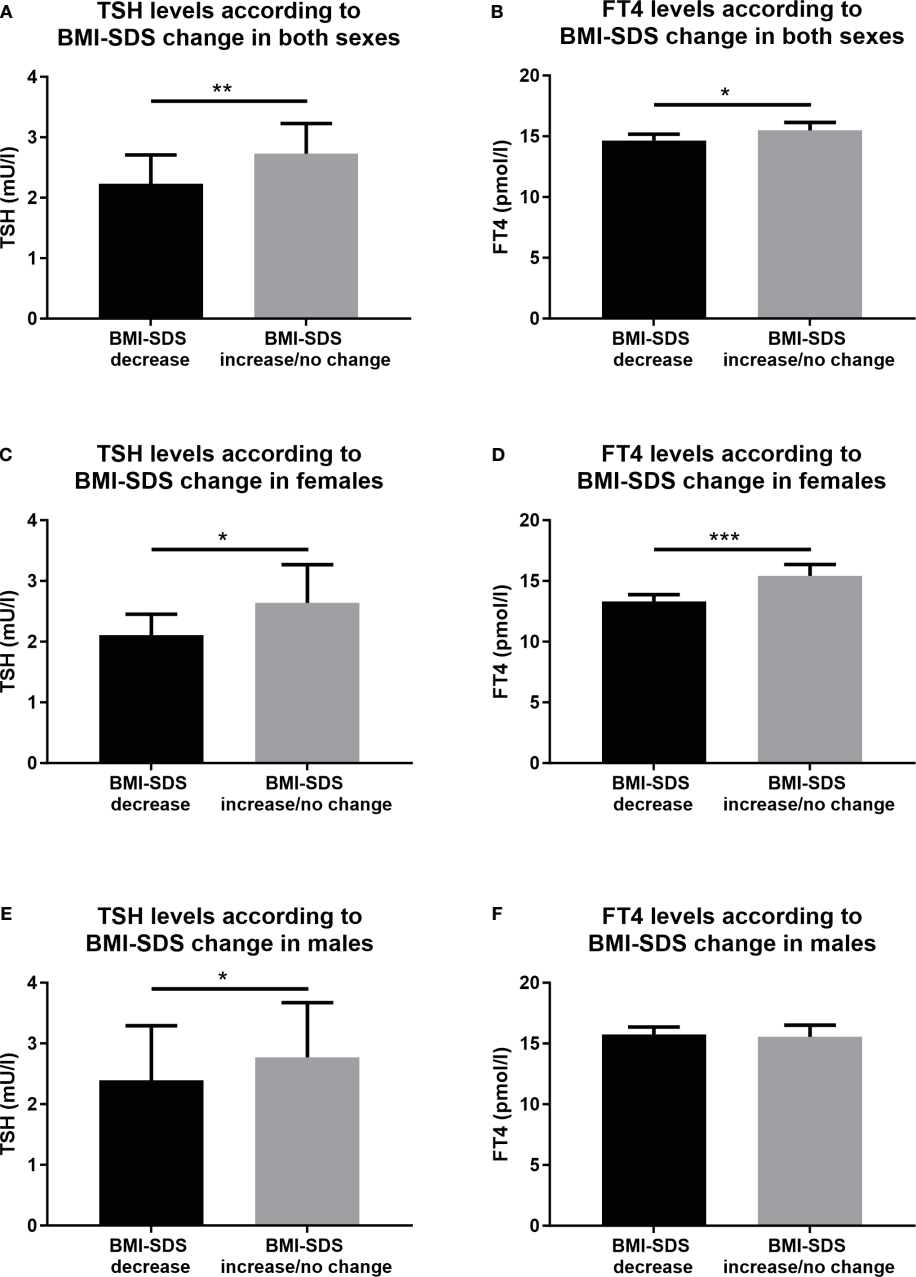
Serum levels of TSH and FT4 in the subgroups of adolescents with obesity according to the BMI-SDS change. **(A)** TSH levels in both sexes, **(B)** FT4 levels in both sexes, **(C)** TSH levels in females, **(D)** FT4 levels in females, **(E)** TSH levels in males, and **(F)** FT4 levels in males. BMI, body mass index; SDS, standard deviation score; TSH, Thyroid-stimulating hormone; FT4, free thyroxine. Included to this analysis were participants from the group of adolescents with obesity. Data for FT4 levels are displayed as mean and 95% confidential intervals for the mean. Data for TSH levels are displayed as median and 95% confidential intervals for the median. Differences were calculated with t-test. TSH data were prior T-test analyses logarithmically transformed. Significant differences were marked with *(p<0.005), **(p<0.01), and ***(p<0.001).

Compared to the group with BMI-SDS increase/no change, adolescents with obesity and a BMI-SDS decrease had significantly lower baseline levels of TSH (median 2.23 and interquartile range of 1.59-3.19 *vs* 2.73; 1.9-3.9 mU/l, p=0.005), and lower baseline levels of FT4 (mean 14.66 ± 2.2 *vs* 15.49 ± 2.74 pmol/l, p=0.048) (**Table 2**; **Figures 1A, B**). No other differences were observed in baseline biochemical and hormonal parameters between the two subgroups of adolescents with obesity (**Table 2**). Food preferences for high sugar, high complex carbohydrate, high protein, high fat and subfractions of fat and low fiber diet were lower in the group with the BMI-SDS decrease, however the differences were not significant (**Table 2**).

**Table 2:** Comparison of the subgroups of adolescents with obesity with BMI-SDS reduction *vs*. increase/no change of the BMI-SDS during the follow up.

Parameter	BMI-SDS increase/no change	BMI-SDS decrease	p
*Baseline data*			
**Age (years)**	16.61 ± 0.89 (71)	16.57 ± 0.97 (73)	0.786
**Sex (% of girls)**	50.7 (73)	43.8 (71)	0.504
**Weight (kg)**	102 ± 25.25 (71)	103.61 ± 17.56 (73)	0.660
**Height (cm)**	172.68 ± 9.42 (71)	173.58 ± 9.62 (73)	0.570
**Height SDS**	0.07 ± 1.18 (71)	0.06 ± 0.97 (73)	0.955
**BMI (kg/m^2^)**	34.08 ± 7.43 (71)	34.29 ± 4.74 (73)	0.841
**BMI SDS**	4.91 ± 2.76 (71)	4.97 ± 1.77 (73)	0.890
TSH (mU/l)*	2.73; 1.9-3.9 (71)	2.23; 1.59-3.19 (71)	**0.005**
**FT4 (pmol/l)**	15.49 ± 2.74 (71)	14.66 ± 2.2 (73)	**0.048**
**FT3 (pmol/l)**	6.09 ± 1.22 (27)	6.24 ± 1.04 (41)	0.607
Fasting serum glucose (mmol/l)*	4.8; 4.5-5.2 (64)	4.8; 4.5-5.1 (64)	0.907
**Urea (mmol/l)**	3.8 ± 1.03 (59)	3.91 ± 0.81 (52)	0.541
**Creatinine (mmol/l)**	62.55 ± 10.52 (65)	64.6 ± 11.04 (72)	0.271
**Uric acid (μmol/l)**	368.8 ± 87.93 (64)	392.29 ± 89.28 (70)	0.128
**Total serum proteins (g/l)**	72.34 ± 4.22 (25)	73.74 ± 5.81 (18)	0.364
AST (μkat/l)*	0.37; 0.32-0.53 (64)	0.4; 0.34-0.5 (68)	0.651
ALT (μkat/l)*	0.43; 0.26-0.62 (66)	0.46; 0.35-0.73 (69)	0.316
GGT (μkat/l)*	0.305; 0.2-0.46 (26)	0.37; 0.28-0.49 (30)	0.537
ALP (μkat/l)*	1.5; 1.27-2 (40)	1.63; 1.33-1.93 (40)	0.557
**Total-cholesterol (mmol/l)**	4.25 ± 1.09 (62)	4.07 ± 0.72 (66)	0.300
**HDL-cholesterol (mmol/l)**	1.14 ± 0.23 (55)	1.11 ± 0.27 (64)	0.477
**LDL-cholesterol (mmol/l)**	2.78 ± 0.82 (54)	2.6 ± 0.64 (64)	0.192
**Triglycerides (mmol/l)**	1.29 ± 0.54 (62)	1.33 ± 0.63 (66)	0.698
**Insulin (mU/l)**	30.67 ± 25.08 (33)	30.58 ± 25.9 (48)	0.987
**High Sugar score**	5.57 ± 1.37 (6)	5.25 ± 1.01 (6)	0.647
**High Complex Carbohydrate score**	6 ± 0.97 (6)	5.35 ± 0.9 (6)	0.259
**High Protein score**	5.6 ± 0.91 (6)	5.23 ± 2.17 (6)	0.710
**High Fat score**	5.5 ± 0.86 (6)	5.41 ± 1.23 (6)	0.882
**Low Fiber score**	5.9 ± 0.9 (5)	5.39 ± 2.05 (4)	0.631
**Score for foods cotaining ≥1.5g of Saturated FA per 100g**	5.55 ± 0.81 (5)	5.35 ± 1.58 (4)	0.805
**Score for foods cotaining ≥1.5g of Polysaturated FA per 100g**	5.6 ± 0.81 (5)	5.47 ± 1.48 (4)	0.869
**Score for foods cotaining ≥1.5g of Monosaturated FA per 100g**	5.68 ± 0.83 (5)	5.35 ± 1.41 (4)	0.674
*Follow-up data*			
**BMI (kg/m2)**	35.59 ± 7.74 (71)	32.63 ± 4.81 (73)	**0.007**
**BMI SDS**	5.46 ± 2.87 (71)	4.33 ± 1.8 (73)	**0.006**
**Duration of the follow-up (years)**	5.52 ± 2.04 (71)	5.16 ± 1.80 (73)	0.271
**BMI change**	1.51 ± 1.5 (71)	-1.66 ± 1.63 (73)	**<0.001**
**BMI-SDS change**	0.55 ± 0.56 (71)	-0.63 ± 0.6 (73)	**<0.001**
**BMI-SDS change per year**	1.27 ± 1.24 (71)	-1.59 ± 1.59 (73)	**<0.001**

*Non-normally distributed data (TSH, fasting serum glucose, AST, ALT, GGT and ALP) are presented as the median and interquartile range, and were log-transformed prior t-test. TSH, Thyroid-stimulating hormone; FT4, free thyroxine; FT3, free triiodothyronine; BMI, body mass index; SDS, standard deviation score; AST, aspartate aminotransferase; ALT, alanine aminotransferase; GGT, gamma-glutamyl transferase; ALP, alkaline phosphatase; FA, fatty acids. Value in parentheses (number of participants). P value was calculated in t-test for the difference between populations of adolescents with obesity and 1. children with obesity and 2. non-obese adolescents. All significant values (p<0.05) are in bold.

We found some differences between adolescent males and females with obesity. In the female group, baseline levels of both TSH and FT4 were significantly lower in the subgroup with the BMI-SDS decrease compared to the subgroup with BMI-SDS increase/no change (2.11; 1.42-2.85 *vs* 2.64; 1.84-3.44 mU/l, p=0.031 for the TSH, and 13.29 ± 1.63 *vs* 15.41 ± 2.77 pmol/l, p<0.001 for the FT4) (**Figures 1C, D**). Whereas in males, only baseline levels of TSH were significantly lower in BMI-SDS decrease group compared to BMI-SDS increase/no change group (2.39; 1.65-3.61 *vs* 2.77; 1.90-4.91 mU/l, p=0.040). There were no significant differences in baseline FT4 levels in both groups of males (15.74 ± 1.99 *vs* 15.56 ± 2.74, p=0.753) (**Figures 1E, F**).

In the groups of children with obesity and non-obese adolescents, no significant differences were found between the BMI-SDS decrease and BMI-SDS increase/no change subgroups regarding the serum baseline levels of TSH (median 2.79; interquartile range 2.12-3.75, *vs*. 3.02; 2.19-4.13 mU/l, p=0.866 in children with obesity and 2.51; 2.15-4.46 *vs* 1.72; 1.44-2.46 mU/l, p=0.071 for non-obese adolescents) and FT4 (mean 15.53 ± 2.68 *vs* 15.37 ± 2.41 pmol/l, p=0.663 for children with obesity and 14.98 ± 1.81 *vs* 16.67 ± 2.33 pmol/l, p=0,114 for non-obese adolescents).

### 3.3 Stratification of the patient population by the levels of TSH and FT4 reveals distinct patients’ characteristics

**Figure 2:**
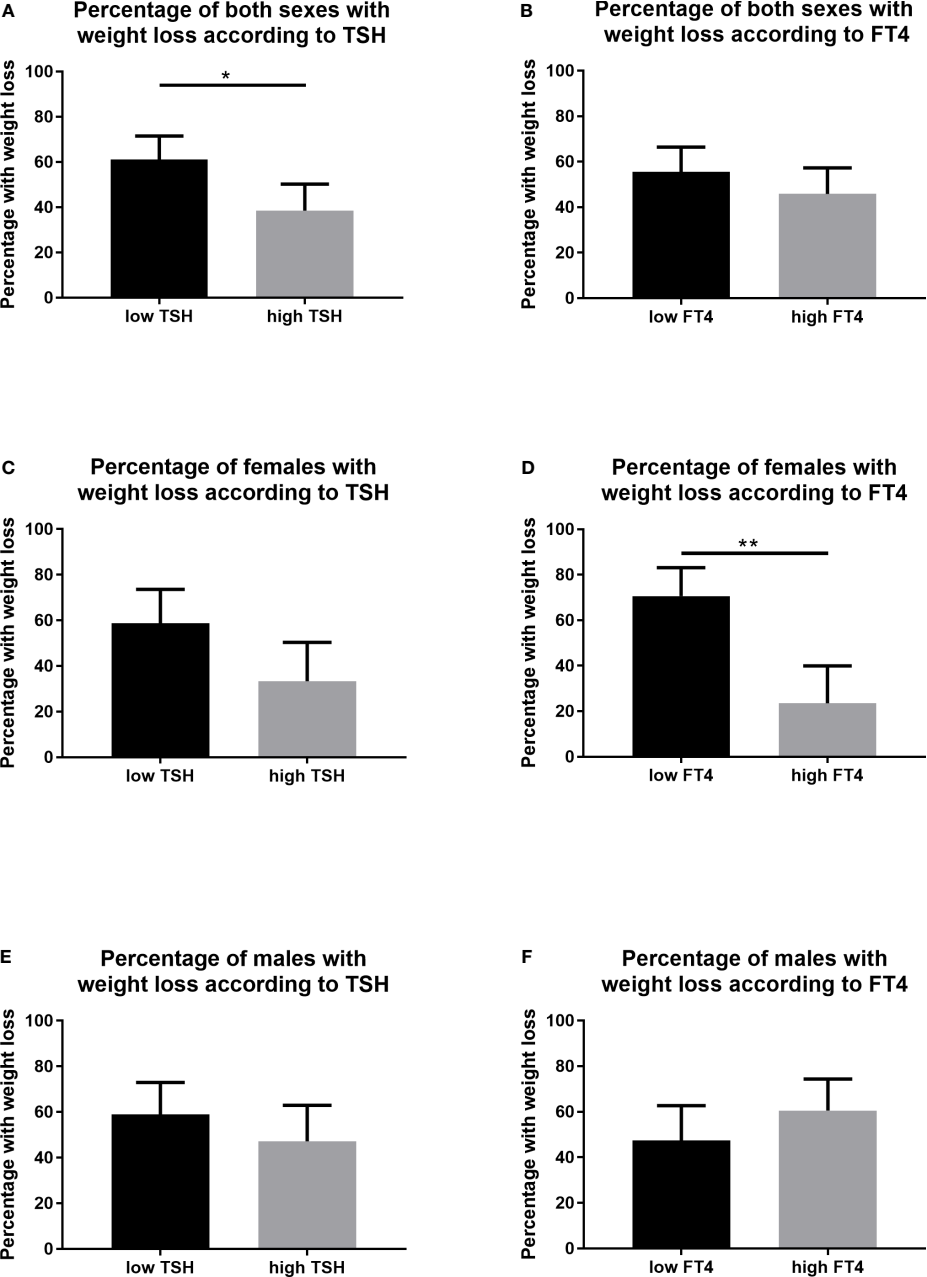
Percentage of participants with BMI-SDS reduction stratified into low and high TSH and FT4 levels. **(A)** TSH levels in both sexes, **(B)** FT4 levels in both sexes, **(C)** TSH levels in females, **(D)** FT4 levels in females, **(E)** TSH levels in males, and **(F)** FT4 levels in males. BMI, body mass index; SDS, standard deviation score; TSH, Thyroid-stimulating hormone; FT4, free thyroxine. Included to this analysis were participants from the group of adolescents with obesity. Stratification in low and high TSH and FT4 subpopulations was made according to the median baseline serum levels of the respective hormones. Confidence intervals for percentages in binary data were calculated using the Wilson/Brown method. Differences were calculated with Fisher’s test for binary data. Significant differences were marked with *(p<0.005), and **(p<0.01).

Stratification of the adolescents with obesity into low TSH and high TSH subgroups by the median baseline serum values revealed significant differences in the percentage of participants who reduced their BMI-SDS in the follow-up. More individuals with the low TSH levels reduced their BMI-SDS compared to the high TSH levels group (61.1, CI: 46.6-71.5 in low TSH vs 38.6; CI: 28.1-50.3% in high TSH group, p=0.012) (**Figure 2A**). Using the same model of stratification for the FT4 baseline serum levels, no significant differences in the percentage of participants with the BMI-SDS reduction were observed (55.6, CI: 44.1-66.5 in low FT4 vs 45.8; CI: 34.8-57.3% in high FT4 group, p=0.317) (**Figure 2B**). In females, no significant differences in the percentage of participants with the BMI-SDS reduction were observed using the stratification for low and high TSH (58.8, CI: 42.2-73.6 vs 33.3; CI: 19.7-50.4%, p=0.051) (**Figure 2C**) . However, significantly higher percentage of adolescent females with obesity reduced their BMI-SDS in the low FT4 group compared to high FT4 group in the follow-up (70.6, CI: 53.8-83.2 in low FT4 vs 23.5; CI: 12.4-40.0% in high FT4 group, p<0.001) (**Figure 2D**). In males, no significant differences in the percentage of participants with the BMI-SDS reduction were observed using the stratification for low and high TSH (60.0, CI: 43.4-72.9 vs 47.2; CI: 40.0-63.0%, p=0.349) (**Figure 2E**), or the stratification for low and high FT4 (47.4, CI: 32.5-62.7 in low FT4 vs 60.5; CI: 44.7-74.4% in high FT4 group, p=0.357) (**Figure 2F**).

Using the same model of stratification for the FT4 baseline serum levels, significantly higher percentage of adolescent females with obesity reduced their BMI-SDS in the low FT4 group compared to high FT4 group in the follow-up (70.6, CI: 53.8-83.2 in low FT4 *vs* 23.5; CI: 12.4-40.0% in high FT4 group, p<0.001) (**Figure 2D**). No significant differences in the percentage of participants with the BMI-SDS reduction were observed in males (47.4, CI: 32.5-62.7 in low FT4 *vs* 60.5; CI: 44.7-74.4% in high FT4 group, p=0.357) (**Figure 2F**) or in both sexes together (55.6, CI: 44.1-66.5 in low FT4 *vs* 45.8; CI: 34.8-57.3% in high FT4 group, p=0.317) (**Figure 2B**).

Stratification of adolescents with obesity into low and high TSH subgroups, and in low and high FT4 subgroups, respectively, revealed no significant differences in the BMI-SDS changes?. However, there was a trend of BMI-SDS reduction per year in the low TSH group and BMI-SDS increase in the high TSH group (-0.41 ± 2.02 *vs* 0.06 ± 2.04 BMI-SDS per year, p=0.167). Similar results were obtained in low FT4 and high FT4 subgroups, as a trend of greater BMI-SDS reduction was seen in the low FT4 group (-0.23 ± 2.05 in low FT4 *vs* -0.13 ± 2.01 BMI-SDS per year in the high FT4 group, p=0.753).

### 3.4 The best predictors of BMI-SDS decrease

Multiple logistic regression analysis aimed to identify the best predictors of variability in the probability of the BMI-SDS reduction in adolescents with obesity encompassed age, sex, baseline BMI-SDS and baseline FT4 and TSH serum levels as covariates. The results revealed that baseline serum TSH levels are the major predictor of variability in the probability of BMI-SDS reduction in males and both sexes together. Baseline serum levels of FT4 were the sole predictor of variability in the probability of BMI-SDS reduction in adolescent females with obesity (**Table 3**).

**Table 3:** Multiple logistic regression analyses of BMI-SDS reduction in adolescents with obesity.

Model No.	Included participants	Model Summary	Dependent	Independent	ΔR^2^	B ± SEM	p value
**1.**	all adolescents with obesity	R^2 =^ 0.077; p = 0.004; n = 142	BMI-SDS reduction	TSH	0.077	-0.34 ± 0.13	**0.009**
**2.**	female adolescents with obesity	R^2 =^ 0.232; p < 0.001; n = 67	BMI-SDS reduction	FT4	0.232	-0.41 ± 0.13	**0.002**
**3.**	male adolescents with obesity	R^2 =^ 0.086; p = 0.025; n = 75	BMI-SDS reduction	TSH	0.086	-0.299 ± 0.15	**0.044**

Analyzed in forward logistic multiple regression analyses. Co-variates: Age, Sex, BMI-SDS, FT4 and TSH. BMI, body mass index; SDS, standard deviation score; TSH, Thyroid-stimulating hormone; FT4, free thyroxine. All significant values (p<0.05) are in bold.

## 4 Discussion

This work clearly shows that BMI-SDS decrease in the 5-month follow-up of adolescents with obesity is associated with lower baseline serum levels of FT4 and TSH. Furthermore, adolescents with obesity and lower baseline TSH levels (stratified by median value) had a 22.5% higher probability (and 61% overall) of reducing their BMI-SDS compared to higher TSH group. In female adolescent group, associations were found with baseline FT4 serum levels (stratified also by median value): participants with lower baseline FT4 levels had a 47.1% higher probability (and 70.6% overall) of reducing their BMI-SDS compared to higher FT4 group. No significant associations of thyroidal hormones with BMI-SDS changes were found in the groups of children with obesity or adolescents without obesity.

### 4.1 Factors underlying the association of thyroid hormones and changes in weight/BMI-SDS

Several studies have analyzed the association of thyroid hormones and changes in body weight (in adults) or BMI-SDS (in children and adolescents) (6). However, there are various factors that can significantly influence results of the studies. Therefore, their results are often conflicting and difficult to compare. Such factors include age of participants. type of intervention, baseline *vs* the change in thyroid hormone levels, and the use of body weight/BMI-SDS data as continuous numeric variable or after transforming to binary one (17). In this study, we used a novel approach analyzing the baseline values with respect to the subsequent weight/BMI-SDS change during the 5-month follow up.

### 4.2 Age

Age is one of the factors that can significantly influence the association of thyroid hormones and weight change/BMI-SDS. While prepubertal children are more controlled by their parents and caregivers, during adolescence they gradually take the initiative in decision making, including the intentional weight reduction in obesity. Patients attitude and behavior regarding lifestyle interventions in obesity thus may be one of the factors that contribute to differences and sometimes inconsistent results seen between children and adults, e.g (18). *vs* (19). Moreover, comparable studies involving exclusively adolescents are lacking. Furthermore, we can speculate that higher psychological maturity in girls may account for sex differences, as in our study where the BMI-SDS association was only seen in adolescent girls, whose results were consistent with those in adults (20).

### 4.3 Natural course studies

Several published longitudinal studies examining associations of thyroid hormones and weight change/BMI-SDS did not have a structured weight reduction intervention during follow-up. It is apparent that studies with a natural course (20) could have different results than those with a targeted structured intervention (18).

Natural development of weight/BMI-SDS under the influence of altered thyroid hormone concentrations can be seen in some thyroid diseases. In primary hypothyroidism characterized by increased TSH and decreased FT4 levels, weight gain may be seen in some patients, although a massive weight gain is rare. In general, 2-4.5 kg (5-10 pounds) of body weight may be attributable to the thyroid, depending on the severity of the hypothyroidism (21). When treated with levothyroxine, only modest weight loss can be seen and this is mediated primarily by loss of excess water and salt rather than fat (22). In children with primary hypothyroidism who are euthyroid on treatment with levothyroxine, a positive association between TSH levels and BMI percentiles was shown (23). On the other hand, hyperthyroidism is traditionally linked to weight loss or even underweight. Most of the studies declare greater impact of hyperthyroidism on body weight when compared to hypothyroidism. Krocker et al. have shown that both hypothyroidism and hyperthyroidism appeared to be associated with alterations in weight and BMI in children. Differences were shown by evaluating associations of weight change with treatment - hyperthyroid patients on treatment gained a mean of 3.4 kg at the first follow-up visit and a mean of 7.1 kg by the second. Contradictory to this, hypothyroid patients lost a minimal amount of weight by the first follow-up (mean of 0.3 kg) and on average gained weight by the second follow-up visit, suggesting that correction of the hyperthyroid state had a greater impact on weight status (24).

TSH and weight/BMI-SDS change. In people without thyroid diseases, most of the longitudinal observational studies in adults have shown, that the weight change during the follow-up is associated with change of the TSH concentration but not with the baseline TSH values (17, 25, 26). Similarly, in our study none of the study groups showed an association of baseline TSH serum levels with the change of BMI-SDS. There could be several explanations of non-significant associations of the thyroid hormones with the weight/BMI-SDS changes. In people without decompensated thyroid disease, changes in thyroid hormone levels are small, mostly in the range of physiological values, and thus, the effect on weight/BMI-SDS should be also small. In individuals with obesity, the situation is even more complex because of the hormonal dysregulation and hormone resistance. Moreover, in several individuals with obesity the changes of weight/BMI-SDS are large, what could misinterpret results. To minimize this effect, we stratified the participants 1. according the BMI-SDS reduction in groups of BMI-SDS increase or decrease, and also calculated the percentage of participants with the BMI-SDS decrease. We found that a BMI-SDS decrease in the follow-up of adolescents with obesity was associated with lower baseline serum levels of TSH, and participants with lower baseline TSH levels (stratified by median value) had a higher probability of reducing their BMI-SDS compared to higher TSH group.

FT4 and weight/BMI-SDS change. As in TSH, there are no studies showing the significant association of baseline FT4 levels with weight/BMI-SDS as continuous variables, only with data transformed to binary variable. In Pizarra study, among individuals without obesity (n=937), those with the higher levels of free triiodothyronine (FT3) or free thyroxine (FT4) had 3-times higher risk of becoming obese during the 6-year follow-up, compared to their counterparts with low thyroid hormone levels (20). We found the same trend in adolescents with obesity, particularly in females. Moreover, our work is the first to find these associations of baseline FT4 and BMI-SDS changes in adolescents with obesity.

### 4.4 Intervention studies

Intervention studies have a structured reduction program that can substantially influence outcomes of the association of thyroid hormones with weight/BMI-SDS changes.

An Italian study with 387 euthyroid adults with obesity who underwent bariatric surgery showed that the individuals with lower TSH levels had a higher percentage of total weight loss, BMI change and a higher percentage of excess weight loss when compared to patients with normal or higher (but still normal) TSH levels (19). On contrary, Wolters et al. (18) have studied the relationships between thyroid hormones and weight status during and 1 year after a 1-year lifestyle intervention in 477 children with obesity. Participants with greater BMI-SDS decrease had higher baseline TSH and FT3 levels compared to children with lower BMI-SDS decrease. On the other hand, children with weight regain 1 year after the end of the intervention had a decrease in TSH and fT3 levels during the intervention compared to children with weight maintenance after the end of the intervention.

In the study by Liu et al. (27) in 579 adults with overweight or obesity and normal thyroid function the authors found that higher levels of baseline FT3 and FT4 were significantly associated with a greater weight loss at 6 months and at 24 months, induced by weight-loss dietary interventions. These data are also in accordance with our results, showing a positive association between FT4 levels and food preferences. Thus, we could speculate that individuals with obesity and higher FT4 levels could profit from weight-loss diets.

### 4.5 Clinical application of the results

Based on our results, adolescents with obesity and higher (than median) baseline TSH and FT4 levels are at higher risk of natural course weight gain/BMI-SDS increase. However, based on results of others, they could benefit from targeted intervention programs (18, 27).

Our data also support the findings from other studies, that treatment with levothyroxine does not decrease weight in individuals with obesity and no thyroid disease (28–31). This could be partially explained by our findings, with higher FT4 levels predisposing weight gainMore complicated is the situation in individuals with obesity and concomitant Hashimoto thyroiditis. Because of TSH resistance, this parameter is useless in monitoring treatment success. Therefore, FT4 would be a better marker and in order to avoid weight gain (as shown by our results), it should be kept in the lower range of normal values.

### 4.6 Limitations

Several factors which were not evaluated in this study, including social and cultural influences, diet, physical activity levels, assessment of energy expenditure and substrate preference, family history and compliance could have an impact on the outcomes. Different duration of the follow-up checks from the baseline (i.e. 5.59 ± 1.85 months) could also influence the results. Also the lack of long-term follow-up limits the understanding of changes over time. Changes in body weight were expressed only by BMI-SDS, as the percentage of fat mass and fat free mass were not evaluated. Also, the values of the thyroid hormones at the follow-up are missing. Moreover, it is a single-center study, and thus further studies in other age groups will be needed to generalize our presumptions.

## 5 Conclusions

Adolescents with obesity who had increased BMI-SDS during the follow-up had significantly higher baseline serum levels of both TSH and FT4. This is the first study to show the association of BMI-SDS change with the baseline FT4 serum levels in adolescents with obesity. These results also confirm the findings from our previous study, linking taste preferences with higher FT4 (12), which might influence weight gain. However, further studies will be needed to generalize our presumptions. Nevertheless, thyroid hormones might represent valuable biomarkers for predicting the weight trajectory in adolescents with obesity.

## Data availability statement

The raw data supporting the conclusions of this article will be made available by the authors, without undue reservation.

## Ethics statement

The studies involving humans were approved by the Ethics Committee of the National Institute for Children´s Diseases in Bratislava, Slovakia. The studies were conducted in accordance with the local legislation and institutional requirements. Written informed consent for participation in this study was provided by the participants’ legal guardians/next of kin.

## Author contributions

DS: Conceptualization, Funding acquisition, Investigation, Methodology, Project administration, Writing – original draft. LK: Investigation, Writing – original draft. DL: Writing – review & editing. EV: Investigation, Writing – review & editing. LT: Investigation, Writing – review & editing. ZP: Investigation, Writing – review & editing. BU: Conceptualization, Funding acquisition, Writing – review & editing. JU: Conceptualization, Formal Analysis, Funding acquisition, Methodology, Writing – review & editing. JS: Conceptualization, Data curation, Formal Analysis, Funding acquisition, Methodology, Project administration, Supervision, Visualization, Writing – review & editing.
